# Intracranial Subdural Hematoma after Spinal Anesthesia for Cesarean Section

**DOI:** 10.1155/2013/253408

**Published:** 2013-12-24

**Authors:** Vittorio Schweiger, Giovanni Zanconato, Gisella Lonati, Silvia Baggio, Leonardo Gottin, Enrico Polati

**Affiliations:** ^1^Department of Anesthesiological Sciences and Specialistic Surgeries, University of Verona, 37134 Verona, Italy; ^2^Department of Life Science and Reproduction, University of Verona, 37134 Verona, Italy; ^3^U.O. di Ginecologia e Ostetricia, Policlinico Borgo Roma, 37134 Verona, Italy

## Abstract

Intracranial subdural hematoma following spinal anesthesia is an infrequent occurrence in the obstetric population. Nevertheless, it is a potentially life-threatening complication. In the majority of the cases, the first clinical symptom associated with intracranial subdural bleeding is severe headache, but the clinical course may have different presentations. In this report, we describe the case of a 38-year-old woman with an acute intracranial subdural hematoma shortly after spinal anesthesia for cesarean section. Early recognition of symptoms of neurologic impairment led to an emergency craniotomy for hematoma evacuation with good recovery of neurologic functions. The possibility of subdural hematoma should be considered in any patient complaining of severe persistent headache following regional anesthesia, unrelieved by conservative measures. Only early diagnosis and an appropriate treatment may avoid death or irreversible neurologic damage.

## 1. Introduction

Spinal and epidural anesthesia have become current standards for obstetric procedures in Italy, both, associated with proven efficacy and safety. Although rare, complications of regional anesthesia do, however, occur, such as intracranial bleeding with subdural hematoma formation [1–3]. Considering the potentially fatal implications, warning signs of intracranial hematoma need to be promptly recognized and adequate treatment started.

We report a case of acute intracranial subdural hematoma following subarachnoid anesthesia for cesarean section, in which appearance of symptoms of early neurological impairment led to emergency craniotomy for hematoma evacuation with good cerebral function recovery. We aim to emphasize the need to carefully assess any obstetric patient complaining of severe headache after regional anesthesia during labor or for a C-section.

## 2. Case Presentation

A 33-year-old nullipara, 38 weeks pregnant, was scheduled for elective cesarean section under spinal anesthesia due to a previous laparoscopic myomectomy. Past medical history was otherwise unremarkable, with no smoking nor alcohol or drug consumption. Routine blood tests, including coagulation status, were normal. After obtaining informed consent and following a 1000 mL i.v. bolus of crystalloid solution, the procedure was performed in the seated position. The subarachnoid space was reached at L3-L4 level at first attempt, using a 25G Sprotte needle, and 10 mg of 0.5% plain bupivacaine administered. The patient was then placed in supine position for C-section. Blood loss during the operation was estimated to be 200 mL and further 500 mL of crystalloids was infused. Hemodynamic parameters were normal and stable during surgery. No vasoactive drug was administered. A healthy, normal baby (male, 2830 g) was delivered with Apgar scores 9 at 1 min and 10 at 5 min.

**Figure 1:**
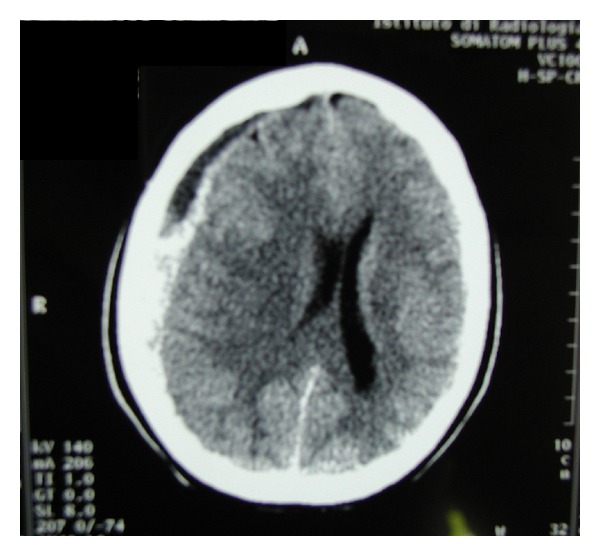
Cranial CT scan showing right sided subdural hematoma.

The clinical course in the early postoperative hours was uneventful. During the 1st day in the obstetric ward 2000 mL of fluids was administered. In the absence of known risk factors for DVT no anticoagulant prophylaxis was given. The following morning the patient complained of nausea without vomiting. While attempting to get out of bed, about 30 hours after surgery, the patient complained of an acute back pain starting from the lumbar region and irradiating upwards along the spine, followed by a severe occipital headache and sudden loss of consciousness. She was brought to the ICU, where she appeared drowsy and bradipnoic. Neurological examination showed anisocoria (right > left). An emergency cranial tomography (CT) revealed an acute subdural hematoma, 12 mm thick, on the convexity of the right hemisphere, with a 10 mm midline shift, compressing the right ventricular system (Figure 1). The patient was transferred to the Neurosurgical Department where a cerebral angiography excluded an associated aneurysm or arteriovenous malformation. An emergency right frontal craniotomy with hematoma evacuation was performed. During the first postoperative day, after withdrawal of sedation, the patient opened her eyes and started to move her hands on command. On the 2nd postoperative day, she showed only a mild right limbs weakness and swelling on her right eyelid. Therapy with oral phenobarbital was started. Two months later the patient was in good clinical condition. She only complained of persistent skull hypoesthesia, numbness at the surgical site, and reduced ability to open her mouth. She did not show any other neurological deficit and was still on antiepileptic drugs six months after surgery.

## 3. Discussion 

Safety of spinal and epidural anesthesia is well documented, and yet serious complications are occasionally related to these procedures. Among them, intracranial subdural hematoma is the most severe and potentially fatal, with a reported incidence of 1/500000 obstetric procedures [4]. However, according to other authors, true prevalence of subdural hematoma is unknown and may be greater than the few published case reports suggest [1, 5].

According to the debut symptoms it may be hard to differentiate a subdural hematoma from postdural puncture headache (PDPH), the most frequent benign complication of spinal anesthesia which improves within a few days if treated with analgesics and bed rest.

Leakage of cerebrospinal fluid (CSF) from the dural hole is the presumed mechanism postulated for PDPH as well as for subdural hematoma. Loss of CSF is believed to lower both intraspinal and intracranial pressures resulting in a caudally directed movement of the spinal cord and brain. Stretching of the pain-sensitive structures and of the intracranial subdural bridging veins occurs. The sudden decrease in the CSF volume may also activate adenosine receptors, thus producing arterial and venous vasodilatation and subsequent clinical symptoms of PDPH. If the traction exerted on the bridging veins is substantial, it may cause a rupture at their weakest point, leading to hematoma formation [6].

Early recognition of intracranial subdural bleeding is crucial to start an effective treatment. Failure to make an early diagnosis of subdural hematoma may result in fatal complications [7, 8].

A subdural hematoma should be suspected when the headache becomes more severe and persistent, even in the recumbent position, in association with neurologic symptoms which include vomiting, blurring of vision, drowsiness, and disorientation. The occurrence of convulsions, diplopia, and high blood pressure after birth may erroneously be interpreted as eclamptic in the absence of an imaging evaluation. Other clinical conditions need also to be ruled out: migraine, meningitis, drug-induced headache (amphetamine and nifedipine), and intracranial pathologies (sinus venous thrombosis, arteriovenous malformations, etc.).

Acute treatment with hypotensive drugs and magnesium sulphate, in case of misdiagnosed dural hematoma, may lead to failure in cerebral autoregulation. Therefore in any patient showing neurological symptoms after spinal or epidural anesthesia, a CT scan should be performed before starting treatment, in order to exclude intracranial bleeding.

Based on the interval between anesthesia and the onset of symptoms, subdural hematomas may be acute and subacute/chronic. Most reported acute cases develop within the first 2 days [1–3, 7, 9, 10] and our patient's case history was typically an acute event: a severe and persistent nonpostural headache, unresponsive to analgesics, with symptoms of acute neurological deterioration, suggesting a sudden increase of intracranial pressure.

While acute bleeding becomes rapidly symptomatic, subacute/chronic subdural bleeding may develop over a period of days or weeks, posing diagnostic problems. A subacute subdural hematoma may act as and be confused with PDPH, causing an initial orthostatic headache, responsive to analgesics, bed rest, and fluid replacement. With time these symptoms may go through alternate phases of improvements and exacerbations, loosing relation with posture and accompanied by neurological signs. According to published studies, interval between dural puncture and recognition of a chronic hematoma is 2 to 4 weeks [5, 8, 11–13]. Since chronic subdural hematomas heal without sequelae if treated timely, a cranial CT is justified if a suspected PDPH does not respond to conservative therapy, increasing in severity or recurring after a pain-free interval [13].

Concerning localization of bleeding, the hematoma may involve the frontal, parietal, and temporal regions (alone or in combination), and although more frequently unilateral, it is not unusual to observe a bilateral intracranial involvement [8, 12, 13].

Acute subdural hematomas are well recognized by a cranial CT scan, whereas chronic intracranial lesions need MRI or cerebral angiography as effective neuroimaging techniques since with time hematoma and surrounding brain tissue show similar radiographic density.

Treatment for subdural hematomas may be surgical or conservative: acute subdural hematomas often cause a rapid neurological deterioration which indicates a surgical evacuation of hematoma by craniotomy or burr holes to reduce the intracranial pressure and preserve brain function. A conservative approach has been recommended for patients with chronic hematoma without mental status changes nor seizure activity, absent intracranial mass effect, and when the hematoma is <1 cm in maximum thickness, causing a midline shift <5 mm [14].

In conclusion, when a patient complains of severe, persistent headache following regional anesthesia unrelieved by conservative measures, one should consider the possibility of subdural hematoma. Careful followup is mandatory in order to come up with an early diagnosis and an appropriate treatment before death or irreversible neurologic damage occurs.
